# Preoperative anemia and associated factors in women undergoing cesarean section at a comprehensive specialized referral hospital in Ethiopia

**DOI:** 10.3389/fmed.2023.1056001

**Published:** 2023-04-04

**Authors:** Alemayehu Eshetu Hassen, Abatneh Feleke Agegnehu, Biruk Adie Admass, Mamaru Mollalign Temesgen

**Affiliations:** ^1^Department of Anesthesia, Dessie Health Science College, Dessie, Ethiopia; ^2^Department of Anesthesia, School of Medicine, College of Medicine and Health Sciences, University of Gondar, Gondar, Ethiopia

**Keywords:** prevalence, cesarean section, preoperative anemia, pregnancy, women

## Abstract

**Background:**

Anemia is a common public health burden during pregnancy. Severe maternal and fetal complications have been associated with anemia. Despite many studies on anemia during pregnancy have been conducted in Ethiopia at any time of antenatal care visits, the prevalence of preoperative anemia among women awaiting cesarean delivery and its contributing factors have not been determined. The aim of this study was to determine the prevalence and associated factors of preoperative anemia in women awaiting cesarean section at a comprehensive specialized hospital in Ethiopia.

**Methods:**

An institution-based cross-sectional study was done from April to June 2022 to determine preoperative anemia in women undergoing cesarean delivery. Data were obtained using a standardized questionnaire that included the women’s background characteristics. Bi-variable and multi-variable logistic regression analyses were performed to identify variables related to preoperative anemia. With a 95% confidence level, the estimated crude odds ratio and adjusted odds ratio were calculated. In a multivariate analysis, variables were considered statistically significant if their p-value was less than 0.05.

**Results:**

A total of 424 pregnant women with a 100% response rate were included in this study. The prevalence of preoperative anemia among women awaiting cesarean delivery was 28.3% (95% CI: 23.8–32.5%). Previous history of abortion, lack of iron supplementation, human immunodeficiency virus infection, previous cesarean section, and American Society of Anesthesiology class III were significantly associated with preoperative anemia among women awaiting cesarean section.

**Conclusion and recommendation:**

Preoperative anemia was diagnosed in a significant proportion of women awaiting cesarean-delivery. Anemia was linked to a lack of iron supplementation, American Society of Anesthesiology class III, previous history of abortion, human immunodeficiency virus infection, and previous cesarean section. Therefore, early detection of high-risk pregnancies, iron supplementation, prevention of HIV infection and due attention to people living with HIV/AIDs are paramount.

## 1. Introduction

Cesarean section is a surgical procedure in which a woman’s abdomen and uterus are opened in order to deliver the fetus. A cesarean section may be indispensable if a vaginal delivery could endanger the mother or the fetus (1). It is one of the most important life-saving operations that helped to lower rates of maternal and neonatal morbidity and mortality.

Cesarean section rates are high and continue to rise (2). One in five women worldwide now give birth via cesarean section. The average cesarean section (CS) rate worldwide is 18.6%, with the rates varying from 6.0 to 27.2% in the least and most developed regions, respectively (3). It is one of the most common operations performed worldwide, with over 23 million performed each year. In low-income countries, cesarean sections account for almost one-third of all surgical procedures performed (4). In Ethiopia, the overall rate of cesarean delivery was 29.55% (5). The rate of cesarean delivery in our setup was 29.7% (6).

Anemia is a global public health problem that affects all physiological groups. Pregnant women are among the most vulnerable populations to anemia, particularly in low-income nations (7). It is defined by the World Health Organization as a low blood hemoglobin concentration (8). It has an impact on individual’s physical and mental health, which has a negative impact on a nation’s economic growth and productivity (8, 9). Anemia is also linked to high maternal and newborn morbidity and mortality especially in poor countries (10).

In spite of the fact that anemia can happen in any population, pregnant women are most commonly exposed to this hematologic disorder (11). According to a WHO report, anemia affects more than half a billion women of reproductive age worldwide. From this, 38% of the anemic women were pregnant (10). Anemia is the most frequent pregnancy related problem, affecting about half of all pregnant women worldwide (12–15).

Anemia is particularly prevalent in underdeveloped countries, where there is a lack of prenatal vitamins, iron, and folic acid intake (8, 12). Iron deficiency anemia is the most frequent type of anemia, affecting mostly women of reproductive age, particularly pregnant women (16, 17). In Ethiopia, the prevalence of anemia among pregnant women was 31.66%. Mothers with short pregnancy interval, consumption of poorly diversified diets, under nutrition, and malaria infection during pregnancy had higher risk to develop anemia (18–20). In our setup, University of Gondar Comprehensive Hospital, the prevalence of anemia among pregnant women attending antenatal care was 22.2% (21).

Anemia is usually caused by the natural physiological changes that occur during pregnancy, which result in low hemoglobin concentration (12, 22). Plasma blood volume expands by around 50% and total red blood cell mass increases by approximately 25% during a single-tone gestation. The greater expansion in plasma is typically reflected by decreases in hemoglobin levels (23, 24).

Anemia is increasingly recognized as a preventable perioperative risk factor, particularly in the obstetric population where postpartum hemorrhage remains a prominent cause of maternal mortality, particularly in low-income countries (25). It is often considered as a risk factor for poor pregnancy outcomes such as premature birth, low birth weight, fetal impairment, and maternal and fetal mortality (11, 26–28). Due to the high prevalence of anemia and the likelihood that they may undergo surgery that results in simultaneous blood loss (such as a cesarean delivery or postpartum hemorrhage), pregnant women are of particular concern (29, 30).

The high prevalence of anemia and rising global rates of cesarean sections make preoperative anemia in pregnant women a serious public health issue (6, 31, 32). Early diagnosis and treatment of preoperative anemia are paramount. Though studies have been conducted on the magnitude of anemia among pregnant women in Ethiopia, none of them had reported the prevalence of anemia among pregnant women awaiting cesarean delivery. Thus, the aim of this study was to determine the magnitude and associated factors of preoperative anemia in women undergoing cesarean section at University of Gondar comprehensive specialized referral hospital in Ethiopia.

## 2. Materials and methods

### 2.1. Study design and setting

An institution-based cross-sectional study was conducted to determine the magnitude and associated factors of preoperative anemia on pregnant women undergoing cesarean section at the University of Gondar comprehensive specialized referral hospital in Ethiopia, from 1 April 2022, to 30 June 2022. The hospital is located in Gondar, a city that has a population of more than five million. The town is situated in the northwest Ethiopian region of Amhara.

### 2.2. Study population, inclusion, and exclusion criteria

Pregnant women who were awaiting cesarean delivery at the University of Gondar comprehensive referral hospital during the data collection period were the study population. All women scheduled for cesarean section during the study period were included, whereas women with no complete blood count, who were seriously ill due to other medical conditions, mentally ill, or unable to speak or hear during the data collection period were excluded.

### 2.3. Operational definition

Anemia in pregnancy is defined as a hemoglobin concentration of less than 11 g/dl. It is considered severe when the hemoglobin concentration is less than 7.0 g/dl, moderate when the hemoglobin falls between 7.0 and 9.9 g/dl, and mild when the hemoglobin is from 10.0 to 10.9 g/dl (33).

*American Society of Anesthesiology (ASA) II*: Normal pregnancy, well-controlled gestational hypertension (HTN), controlled preeclampsia without severe features, diet-controlled gestational diabetes mellitus (5) (34).

*American Society of Anesthesiology (ASA) III*: Preeclampsia with severe features, gestational DM with complications or high insulin requirements, a thrombophilic disease requiring anticoagulation (34).

*Antenatal care (9) follow-up*: Antenatal care follow-up was defined as the self-reported frequency of any of the ANC services delivered by skilled health staff at the health facility. If the pregnant woman had one or more ANC visits, she is considered to have had ANC follow-up; otherwise, she is considered to have had no history of ANC follow-up (35).

*Iron and folic acid supplement*: If a pregnant woman receives and consumes a standard dose of 30–60 mg of iron and 400 μg of folic acid daily, starting as early as possible and continuing throughout pregnancy, she is considered to have optimal iron and folic acid supplementation (36).

*History of abortion*: A woman is considered to have a history of abortion if she terminated her pregnancy before the age of viability, which is 20 and 28 weeks gestational age for developed and developing countries, respectively, either spontaneously or by induction (37).

### 2.4. Sample size and sampling procedures

#### 2.4.1. Sample size determination

The sample size of the study was determined by using a single population proportion formula. The size of the study participants was calculated using a 95% level of confidence and a 5% margin of error.

Although the prevalence of anemia during pregnancy was determined in pregnant women at any antenatal care (9) visit, no study was conducted in Ethiopia to determine the magnitude of preoperative anemia in women awaiting cesarean delivery. As a result, the 0.5 proportion assumption was applied. With a 10% non-response rate, the final sample size was 424.

#### 2.4.2. Sampling technique

Simple random sampling technique was used to select the study participant. Participants who fulfilled the inclusion criteria were randomly selected and included in the study until the required sample size was achieved.

#### 2.4.3. Data collection procedure

A structured questionnaire was prepared by the principal investigator. A pretested interviewer-based questionnaire was used to collect data, which included socio-demographic characteristics as well as health and dietary factors of pregnant women undergoing cesarean section. Obstetrics and medical history of women were collected from the chart.

#### 2.4.4. Data quality control

The lead investigator offered training to data collectors. A pre-test on 10% of pregnant women who were not involved in the main study was undertaken to assure data quality. The questionnaire for the main study received the necessary modifications. Important information regarding the questionnaire was provided to the data collectors. The collected data was reviewed by the lead investigator for completeness and accuracy.

#### 2.4.5. Data processing and analysis

Epi-Data version 4.6 was used to enter the data, which was then exported to SPSS version 20 for analysis. Socio-demographic variables of the women were analyzed and presented in tables, a pie chart, and narrations. The data were presented by median and interquartile range. Variables with a p-value of less than 0.2 in binary logistic regression were transferred to multivariable logistic regression, and variables with a p-value of less than 0.05 with a 95% confidence interval were deemed predictor variables for preoperative anemia in women awaiting cesarean section.

#### 2.4.6. Ethical consideration

Ethical clearance was obtained from the ethical review committee of the school of medicine, at University of Gondar. Written informed consent was obtained from each study participant after a clear explanation of the study. Anyone who refused to participate in the study was informed that they had the complete right to withdraw at any time. The data collected from participants was kept confidential.

## 3. Results

### 3.1. Socio-demographic characteristics of women undergoing cesarean section

The study included 424 pregnant women with a 100% response rate. The median age of the study population was 28 years old, with an IQR of 24–31 years. A total of 222 (52.4%) of the participants were between the ages of 26 and 33. Out of 424 participants, 275 (64.9%) lived in urban regions; whereas the remaining 149 (35.1%) were from rural areas. More than half of the study participants, 218 (51.4%), were housewives, while 113 (26.7%) were government employees (Table 1).

**TABLE 1:** Socio-demographic characteristics of women undergoing cesarean section at University of Gondar comprehensive specialized hospital, 2022.

Variables	Category	Preoperative anemia		Frequency (%)
		Yes	No	
Age	18–25 years	33 (22.4%)	114 (77.6%)	147 (34.7%)
	26–34 years	69 (31.1%)	153 (68.9%)	222 (52.3%)
	>34 years	18 (32.7%)	37 (67.3%)	55 (13.0%)
Marital status	Single	8 (34.8%)	15 (65.2%)	23 (5.4%)
	Married	105 (27.6%)	276 (72.4%)	381 (89.9%)
	Other	7 (35.0%)	13 (65.0%)	20 (4.7%)
Occupation	house wife	79 (36.2%)	139 (63.8%)	218 (51.4%)
	Government employed	20 (17.7%)	93 (82.3%)	113 (26.7%)
	privately employed	7 (36.8%)	12 (63.2%)	19 (4.5%)
	Daily laborer	8 (28.6%)	20 (71.4%)	28 (6.6%)
	Merchant	6 (13.0%)	40 (87.0%)	46 (10.8%)
Level of education	Cannot write and read	42 (54.5%)	35 (45.5%)	77 (18.2%)
	Primary education	33 (38.8%)	52 (61.2%)	85 (20.0%)
	Secondary education	18 (15.9%)	95 (84.1%)	113 (26.7%)
	College and above	27 (18.1%)	122 (81.9%)	149 (35.1%)
Residence	Urban	42 (15.3%)	233 (84.7%)	275 (64.9%)
	Rural	78 (52.3%)	71 (47.7%)	149 (35.1%)

### 3.2. Clinical and nutritional characteristics of women undergoing cesarean section

The majority, 314 (74.1%), of women had heard about anemia, while 115 (27.1%) had a history of malaria. The majority, 383 (90.3%), of respondents had meat and animal products in their diet for the last three months. About 303 (71.5%) women had emergency cesarean sections, while 121 (28.5%) underwent elective cesarean sections (Table 2).

**TABLE 2:** Clinical and obstetric characteristics of women undergoing cesarean section at University of Gondar comprehensive specialized hospital, 2022.

Variables	Category	Preoperative anemia		Frequency (%)
		Yes	No	
Types of CS	Elective	19 (15.7%)	102 (84.3%)	121 (28.5%)
	Emergency	101 (33.3%)	202 (66.7%)	303 (71.5%)
Folic acid intake	No	106 (33.9%)	207 (66.1%)	313 (73.8%)
	Yes	14 (12.6%)	97 (87.4%)	111 (26.2%)
Multivitamin intake	No	114 (30.2%)	263 (69.8%)	377 (88.9%)
	Yes	6 (12.8%)	41 (87.2%)	47 (11.1%)
Green leafy vegetables in diet	No	11 (57.9%)	8 (42.1%)	19 (4.5%)
	Yes	109 (26.9%)	296 (73.1%)	405 (95.5)
Meat and animal products in diet	No	16 (39.0%)	25 (61.0%)	41 (9.7%)
	Yes	104 (27.2%)	279 (72.8%)	383 (90.3%)
Had fruit after meal	No	30 (41.1%)	43 (58.9%)	73 (17.2%)
	Yes	90 (25.6%)	261 (74.4%)	351 (82.8%)
Heard about anemia	No	29 (26.4%)	81 (73.6%)	110 (25.9%)
	Yes	91 (29.0%)	223 (71.0%)	314 (74.1%)
History of malaria	No	86 (27.8%)	223 (72.2%)	309 (72.9%)
	Yes	34 (29.6%)	81 (70.4%)	115 (27.1%)
Diabetes mellitus	No	113 (27.9%)	292 (72.1%)	405 (95.5%)
	Yes	7 (36.8%)	12 (63.2%)	19 (4.5%)

### 3.3. Magnitude of preoperative anemia in women undergoing cesarean section

**FIGURE 1:**
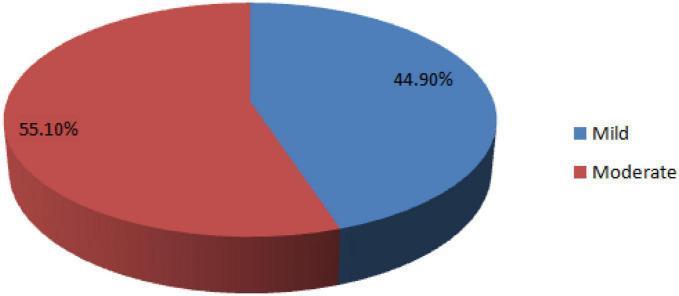
Severity of preoperative anemia in women undergoing cesarean section at University of Gondar comprehensive specialized hospital, 2022.

The hemoglobin range of the women was 7.9–14.4 g/dl. The overall prevalence of preoperative maternal anemia was 28.3% (95% CI: 23.8–32.5%). Of the anemic women, 55.1 and 44.9% were moderately and mildly anemic, respectively (Figure 1).

### 3.4. Risk factors associated with preoperative anemia in women undergoing cesarean delivery

In the bi-variable logistic regression, variables such as residence, ANC follow-up, iron supplementation, ASA status, HIV/AIDS, malignancy, respiratory disease, renal disease, hypertension, body mass index, gravidity, abortion, and previous cesarean sections were found to be potential predictor of anemia (*P* < 0.2) and fitted for the final analysis model.

### 3.5. Multi-variable analysis of factors associated with maternal preoperative anemia

In the final multivariable logistic regression analysis, ASA physical status, previous number of cesarean sections, history of abortion, HIV/AIDS, and lack of iron supplement were significantly associated with preoperative anemia in women undergoing cesarean section (Table 3).

**TABLE 3:** Multivariable logistic regression analysis of associated factors of preoperative anemia among women undergoing CS at University of Gondar comprehensive specialized referral hospital, 2022 (*N* = 424).

Variable	Category	Preoperative anemia		Odds ratio (95% CI)		*P*-value
		Yes	No	COR	AOR	
Iron supplement	No	54 (76.1%)	17 (23.9%)	13.8	4.2 (1.124–15.747)	0.033*
	Yes	66 (18.7%)	287 (81.3%)	1	1	
Abortion	No	59 (18.4%)	262 (81.6%)	1	1	
	Yes	61 (59.2%)	42 (40.8%)	13.2	9.95 (4.566–21.676)	0.001*
HIV infection	No	85 (22.8%)	278 (77.2%)	1	1	
	Yes	35 (67.3%)	17 (32.7%)	6.95	3.33 (1.230–9.005)	0.018*
Previous CS	No CS	16 (10.3%)	139 (89.7%)	1	1	
	One CS	55 (30.1%)	128 (69.9%)	3.73	2.8 (1.192–6.662)	0.018*
	≥ 2 CS	49 (57.0%)	37 (43.0%)	11.5	3.9 (1.375–11.099)	0.011*
ASA status	ASA II	73 (20.8%)	278 (79.2%)	1	1	
	ASA III	47 (64.4%)	26 (35.6%)	6.88	3.25 (1.155–9.146)	0.026*

1: Reference. *: significant. AOR, adjusted odds ratio; COR, crude odds ratio; CI, confidence interval; ASA, American Society of Anesthesiologist; HIV, human immunodeficiency virus; CS, cesarean section.

The odds of developing preoperative anemia were 9.95 times greater in women with an abortion history (CI: 4.566–21.676) than in women without any abortion history. Women who did not get iron supplementation (CI: 1.124–17.447) were 4.2 times more likely to have preoperative anemia than women who received iron supplementation. Women with HIV (CI: 1.230–9.005) were 3.33 times more likely to develop preoperative anemia than those without HIV infection (Table 3).

The likelihood of developing preoperative anemia among women who had one previous cesarean section (CI: 1.192–6.662) was 2.8 times higher than that of a woman with no previous cesarean scar. Women with two or more previous cesarean sections (CI 1.375–11.099) were 3.9 times more likely to have preoperative anemia when compared to mothers who had no previous cesarean section. Women with ASA III physical status were 3.25 times more likely to be anemic than those with ASA II physical status (CI: 1.155–9.146) (Table 3).

## 4. Discussion

In this study, the proportion of anemic women awaiting cesarean delivery was 28.3% and remained a moderate public health problem. This suggests that preoperative anemia remains an issue in this segment of population. Unless appropriate initiatives are established and implemented, the problem will affect the country’s effort in lowering maternal and child mortality rate.

The prevalence of preoperative anemia in our study was higher than the findings of studies conducted in Turkey (9.7%) (38) and China (16.6%) (25). These variations could be due to socioeconomic factors, geographic location, or the quality of healthcare services. However, the proportion of anemia in this study was lower than in studies done in India (47.9%) (39). The reason for this discrepancy might be attributed to a difference in sample size.

The multivariable logistic regression analysis of this study revealed that ASA physical status, lack of iron supplementation, previous history of abortion, HIV infection, and previous cesarean section were significantly associated with preoperative anemia in women undergoing cesarean section.

Women who had previous cesarean deliveries were about three times more likely to have preoperative anemia than women who had no previous cesarean sections. The study conducted in Taiwan showed that women with a higher number of prior cesarean sections were more likely to be anemic (40). Excessive bleeding after CS is common and contributes to the development of anemia (41). This could be explained by the fact that repeated cesarean sections during reproductive age are a cause of anemia later in life. This might be because the average amount of blood loss caused by a cesarean section is approximately twice that of a vaginal delivery. For nearly 25 years, this excessive blood loss may not be compensated (42). Furthermore, recovery in physical activity performance is faster in women after vaginal delivery than in those following cesarean delivery since a cesarean section produces more pain, requires more time to recover from anesthesia, and causes more morbidities. Reduced activity has been identified as a risk factor for anemia (43, 44).

Preoperative anemia was four times more likely among women who did not take iron supplements during their pregnancy than in women who did. This report was consistent with studies conducted in Ethiopia (31, 45). This may be due to pregnant women taking their iron supplements, which can help them raise their hemoglobin levels and prevent anemia during pregnancy. Since pregnancy is a highly iron-demanding period due to the increased iron needs to supply the expanding blood volume of the mother and the rapidly growing fetus and placenta, pregnant women are most likely to develop anemia if they didn’t receive iron supplements during pregnancy (45). During the final phase of pregnancy, maternal iron stores are depleted, and the requirement for iron cannot be easily met by diet alone. As a result, the risk of iron deficiency anemia is considerable, particularly at the end of pregnancy (46).

Our study found that pregnant women with HIV were three times more likely to have preoperative anemia than HIV-negative pregnant women. Cross-sectional studies conducted in Ethiopia and Nigeria indicated a substantial relationship between HIV/AIDS and anemia and documented HIV as a cause of anemia in pregnancy, which is consistent with our findings (47–49). This increased risk of anemia among HIV-positive pregnant women may be explained by the reality that HIV infection is related to lower levels of serum folate and serum ferritin. It is also possible that HIV infection causes anemia through changes in cytokine production, altered erythropoietin response to bone marrow, and the utilization of antiretroviral drugs, particularly zidovudine (50, 51).

Women with ASA III physical status were three times more likely to develop preoperative anemia than those with ASA II physical status. The phenomenon may be explained by the fact that patients with higher ASA classification tend to have more severe comorbidities and chronic illnesses (50, 52).

The odds of having preoperative anemia among women who had an abortion history were about ten times higher than among women who had not an abortion history. In agreement with our finding, a cross-sectional study carried out in Ethiopia stated that women who had previous abortions were more likely to have anemia (53). This could be due to significant blood loss, which reduces stored iron while increasing the body’s need for iron. Women who have had an abortion in the past are at risk of anemia (54).

## 5. Strength and limitation

The prevalence and variables related to preoperative anemia in women awaiting cesarean delivery were investigated in this study. It will serve as a foundation for future research in the study region and elsewhere.

This study, however, did not identify the type of anemia or the underlying causes based on cytological data. This study cannot demonstrate a causal association between the outcome and explanatory variables because it used a cross-sectional study design. In addition, the women’s dietary diversity was not assessed using 24 h food recall method.

## 6. Conclusion and recommendation

The overall proportion of preoperative anemia in pregnant women before cesarean section remained a public health problem. Lack of iron supplementation, ASA III physical status, and previous history of abortion, HIV infection, and previous cesarean section were found to be significantly associated with preoperative anemia in pregnant women awaiting cesarean delivery.

Therefore, early detection of high-risk pregnancies, iron supplementation, prevention of HIV infection and due attention to people living with HIV/AIDs are paramount. We also suggest that future researchers to conduct a study on the outcome of a preoperative anemia in women who underwent cesarean section.

## Data availability statement

The raw data supporting the conclusions of this article will be made available by the authors, without undue reservation.

## Ethics statement

The studies involving human participants were reviewed and approved by the School of Medicine, College of Medicine and Health Sciences, University of Gondar. The patients/participants provided their written informed consent to participate in this study.

## Author contributions

All authors contributed to the study’s idea and design, data collection, analysis, and interpretation, manuscript drafting, and final approval of the manuscript.
